# Clinical and Laboratory Factors Associated With In-Hospital Mortality Among Patients With Decompensated Liver Cirrhosis: A Retrospective Study From Indonesia

**DOI:** 10.7759/cureus.110443

**Published:** 2026-06-08

**Authors:** Hendra Raharja, Taufik Sungkar, Ilhamd Ilhamd

**Affiliations:** 1 Department of Internal Medicine, Faculty of Medicine, Universitas Sumatera Utara, Medan, IDN

**Keywords:** child-pugh class, decompensated liver cirrhosis, hepatic encephalopathy, hospitalized patients, in-hospital mortality, meld-na score

## Abstract

Background: Liver cirrhosis is a major cause of morbidity and mortality worldwide. Identification of clinical and laboratory factors associated with mortality may help clinicians identify high-risk hospitalized patients with decompensated liver cirrhosis.

Methods: A retrospective cohort study was conducted among 190 patients with decompensated liver cirrhosis admitted to Adam Malik General Hospital, Medan, Indonesia, between January 2022 and December 2023. Demographic, clinical, and laboratory data were obtained from medical records. Bivariate analyses were performed using chi-square, Fisher's exact, and Mann-Whitney U tests as appropriate.

Results: Among the 190 patients included in the study, 79 (41.6%) died during hospitalization. Bivariate analysis showed that grade III-IV hepatic encephalopathy, abnormal leukocyte count, low serum albumin levels, elevated total bilirubin, elevated serum creatinine, increased INR, hyponatremia, and higher albumin-bilirubin (ALBI) grade, Child-Pugh class, model for end-stage liver disease (MELD), and MELD sodium (MELD-Na) scores were significantly associated with in-hospital mortality (p < 0.05). In contrast, age, sex, comorbidities, and upper gastrointestinal bleeding at admission were not significantly associated with mortality.

Conclusion: In-hospital mortality among decompensated cirrhotic patients remains high. Several clinical and laboratory parameters, including hepatic encephalopathy, leukocyte count, albumin, bilirubin, creatinine, INR, and sodium levels, were significantly associated with mortality. Early recognition of these factors may help clinicians identify high-risk patients with decompensated liver cirrhosis during hospitalization.

## Introduction

Liver cirrhosis represents the end stage of chronic liver disease, characterized by progressive hepatic fibrosis and distortion of the liver architecture, resulting in impaired liver function. Liver cirrhosis remains a major global health problem and contributes substantially to morbidity and mortality worldwide. According to the Global Burden of Disease study, liver cirrhosis accounted for approximately 1.47 million deaths globally in 2019, with Southeast Asia carrying one of the highest disease burdens [1].

In Indonesia, liver cirrhosis is frequently found in individuals of productive age and often presents at the decompensated stage, therefore increasing the risk of in-hospital mortality [2]. Clinical factors, such as hepatic encephalopathy, ascites, upper gastrointestinal bleeding, and laboratory parameters including albumin, creatinine, bilirubin, international normalized ratio (INR), and serum sodium, have been recognized as factors associated with mortality among patients with liver cirrhosis [3-5]. However, findings may differ across populations due to differences in epidemiology, etiologies of liver disease, and access to healthcare providers.

This study aimed to evaluate clinical and laboratory factors associated with in-hospital mortality among patients with decompensated liver cirrhosis admitted to Adam Malik General Hospital, Medan. The findings may provide local evidence regarding factors associated with mortality in this population.

## Materials and methods

This was a retrospective cohort study conducted at Adam Malik General Hospital, Medan, North Sumatra, Indonesia. Data were obtained from the medical records of patients hospitalized between January 2022 and December 2023. This study was approved by the Health Research Ethics Committee of the Faculty of Medicine, Universitas Sumatera Utara, under the registration number of 332/KEPK/USU/2025.

Study population

The study population included hospitalized patients with decompensated liver cirrhosis during the study period. The inclusion criteria were patients aged ≥ 18 years with a diagnosis of decompensated cirrhosis established by clinical, laboratory, and/or imaging findings. All included patients presented with ascites during hospitalization. This was not an inclusion criterion; rather, all eligible hospitalized patients with decompensated liver cirrhosis identified during the study period were found to have ascites. Exclusion criteria included incomplete medical records and patients with a history of liver transplantation.

Variables

Collected variables included demographic data (age, sex), clinical characteristics (upper gastrointestinal bleeding at admission, hepatic encephalopathy, comorbidities), and laboratory parameters (leukocyte count, albumin, total bilirubin, serum creatinine, INR, and serum sodium), including Child Pugh score, albumin-bilirubin (ALBI) grade, model for end-stage liver disease (MELD)/MELD-sodium (MELD-Na) score. Upper gastrointestinal bleeding at admission was defined as the presence of hematemesis and/or melena documented in the medical record. Leukocyte count was categorized as normal or abnormal according to the institutional laboratory reference range. The abnormal category included both leukopenia and leukocytosis. The primary outcome was in-hospital mortality, defined as death occurring during the index hospitalization. 

Statistical analysis

Statistical analysis was performed using Statistical Product and Service Solutions (SPSS, version 25.0; IBM SPSS Statistics for Windows, Armonk, NY). Numerical variables were tested for normality using the Kolmogorov-Smirnov test. Because total bilirubin, serum creatinine, and INR showed markedly skewed distributions, log-transformed values were used for statistical comparisons, while descriptive statistics were reported using the original clinical values. Normally distributed variables were presented as mean ± standard deviation, while non-normally distributed variables were presented as median (minimum-maximum). Categorical variables were expressed as frequencies and percentages. Bivariate analysis was performed to evaluate factors associated with in-hospital mortality. Chi-square test or Fisher's exact test was used for categorical variables as appropriate, while the Mann-Whitney U test was used for non-normally distributed numerical variables. A p-value < 0.05 was considered statistically significant.

## Results

A total of 190 patients with liver cirrhosis were included in this study. Among them, 79 (41.6%) died during hospitalization, while 111 (58.4%) survived until discharge. Baseline characteristics showed that the mean age was 54.9 years, with a predominance of male patients. Most patients (130, 68.4%) had no comorbidities. Upper gastrointestinal bleeding at admission was observed in 104 (54.7%) patients, while 109 (57.4%) had no hepatic encephalopathy. Among patients who died during hospitalization, 51 (64.6%) presented with grade III-IV hepatic encephalopathy. All 190 patients (100%) had ascites during hospitalization. For full information, please refer to Table 1.

**Table 1:** Baseline characteristics and factors associated with in-hospital mortality among patients with decompensated liver cirrhosis SD: standard deviation; INR: international normalized ratio; ALBI: albumin-bilirubin score; MELD: model for end-stage liver disease; MELD-Na: model for end-stage liver disease-sodium Categorical variables were compared using Pearson's chi-square test or Fisher's exact test, as appropriate. Continuous variables were compared using the Mann-Whitney U test. For total bilirubin, serum creatinine, and INR, statistical comparisons were performed using log-transformed values because of non-normal distributions.

Characteristics	Deceased (n = 79)	Alive (n = 111)	Total (n = 190)	Test Statistics	p-value
Age (years), mean ± SD	55.73 ± 10.26	54.37 ± 9.59	54.94 ± 9.87	U = 4058.0	0.382
Male, n (%)	64 (81.0)	82 (73.9)	146 (76.8)	χ² = 1.322	0.250
Female, n (%)	15 (19.0)	29 (26.1)	44 (23.2)		
Comorbidity, n (%)	22 (27.8)	38 (34.2)	60 (31.6)	χ² = 0.871	0.351
Upper gastrointestinal bleeding, n (%)	46 (58.2)	58 (52.3)	104 (54.7)	χ² = 0.665	0.415
No hepatic encephalopathy, n (%)	20 (25.3)	89 (80.2)	109 (57.4)	χ² = 75.954	< 0.001
Hepatic encephalopathy grade I – II, n (%)	8 (10.1)	15 (13.5)	23 (12.1)		
Hepatic encephalopathy grade III – IV, n (%)	51 (64.6)	7 (6.3)	58 (30.5)		
Normal leukocyte count (x 10^3^/µL), n (%)	29 (36.7)	73 (65.8)	102 (53.7)	χ² = 15.672	< 0.001
Abnormal leukocyte count (x 10^3^/µL), n (%)	50 (63.3)	38 (34.2)	88 (46.3)		
Serum albumin (g/dL), mean ± SD	2.36 ± 0.54	2.61 ± 0.56	2.51 ± 0.56	U = 3310.5	0.004
Total bilirubin (mg/dL), median (min-max)	3.7 (0.20-42.0)	1.32 (0.22-14.4)	2.17 (0.20-42.0)	U = 2529.0	< 0.001
Serum creatinine (mg/dL), median (min-max)	2.41 (0.52-16.9)	1.08 (0.37-10.3)	1.3 (0.37-16.9)	U = 2231.0	< 0.001
INR, median (min-max)	1.65 (0.88-5.1)	1.35 (0.59-3.2)	1.45 (0.59-5.1)	U = 2470.0	< 0.001
Serum sodium (mEq/L), median (min-max)	132 (112-151)	139 (119-158)	136 (112-158)	U = 3022.0	0.001
ALBI grade 1, n (%)	0 (0)	4 (3.6)	4 (2.1)	χ² = 16.026	< 0.001
ALBI grade 2, n (%)	16 (20.3)	49 (44.1)	65 (34.2)		
ALBI grade 3, n (%)	63 (79.7)	58 (52.3)	121 (63.7)		
Child-Pugh class A, n (%)	0 (0)	2 (1.8)	2 (1.1)	χ² = 37.129	< 0.001
Child-Pugh class B, n (%)	11 (13.9)	62 (55.9)	73 (38.4)		
Child-Pugh class C, n (%)	68 (86.1)	47 (42.3)	115 (60.5)		
MELD, median (min-max)	24 (7-40)	13 (6-36)	16.5 (6-40)	U = 1629.5	< 0.001
MELD-Na, median (min-max)	23 (10-40)	14 (7-33)	17 (7-40)	U = 1662.5	< 0.001

Bivariate analysis (Table 1) revealed that several clinical and laboratory parameters were significantly associated with in-hospital mortality. Grade III-IV hepatic encephalopathy and abnormal leukocyte count were significantly associated with mortality (both p < 0.001).

Low serum albumin levels were significantly associated with higher mortality (p = 0.004). Elevated total bilirubin, elevated serum creatinine, increased INR, and low serum sodium levels were also significantly associated with mortality (all p ≤ 0.001).

Furthermore, higher ALBI grade, Child-Pugh class, MELD score, and MELD-Na score were significantly associated with in-hospital mortality (all p < 0.001). In contrast, age (p = 0.382), sex (p = 0.250), comorbidities (p = 0.351), and upper gastrointestinal bleeding at admission (p = 0.415) were not significantly associated with in-hospital mortality.

## Discussion

This study demonstrated a relatively high in-hospital mortality rate of 41.6% among hospitalized patients with decompensated liver cirrhosis. Compared to a previous Indonesian study conducted at Cipto Mangunkusumo Hospital, which reported an in-hospital mortality rate of 12.03% [2], the mortality observed in our cohort was substantially higher. One possible explanation is that all patients included in this study had ascites, indicating that the study population predominantly consisted of patients with decompensated cirrhosis. Notably, ascites was not used as a selection criterion; however, all eligible patients identified during the study period presented with ascites, reflecting the predominance of advanced decompensated cirrhosis in our hospitalized cohort. In addition, a considerable proportion of patients presented with advanced hepatic encephalopathy and high Child-Pugh or MELD scores, reflecting severe disease at admission. These findings suggest that disease severity and late presentation may have contributed substantially to the observed mortality rate.

These findings are also consistent with global data showing that decompensated cirrhosis is associated with high short-term mortality, particularly among hospitalized patients with advanced disease and organ dysfunction [6]. The differences that had been found may reflect variations in patient characteristics, particularly the predominance of advanced or decompensated cirrhosis at admission, as well as differences in healthcare access and referral patterns.

Several factors were significantly associated with mortality in this study, including grade III-IV hepatic encephalopathy, low serum albumin, elevated creatinine, elevated bilirubin, abnormal leukocyte count, high INR, and low serum sodium. Hepatic encephalopathy is a common complication of cirrhosis, with a prevalence of 30-45% [7], and is widely recognized as a factor associated with both short- and long-term mortality. Our findings are consistent with Bjerring et al., who reported that 90-day mortality was significantly higher in patients with grade III-IV hepatic encephalopathy compared to those with lower grades or none [3]. This is in line with current understanding that acute decompensation and organ failure, including hepatic encephalopathy, are major determinants of short-term mortality in cirrhosis [6].

Serum albumin reflects both hepatic synthetic function and nutritional status. In this study, hypoalbuminemia was significantly associated with increased mortality, consistent with prior reports showing that albumin < 3.5 g/dL is linked to reduced five-year survival among cirrhotic patients [8]. In addition, albumin plays important roles beyond oncotic pressure, including anti-inflammatory and immunomodulatory functions, which may explain its strong association with clinical outcomes in decompensated cirrhosis [9].

An important finding in this study is the significant association between abnormal leukocyte count and mortality. This may indicate the presence of systemic inflammation or infection, which is common in cirrhotic patients. Systemic inflammatory response syndrome and bacterial infections are well-recognized contributors to acute decompensation and mortality in cirrhosis. Elevated leukocyte levels may reflect ongoing inflammatory processes that contribute to multi-organ dysfunction [10]. However, specific infectious complications were not evaluated in the present study. Therefore, the observed association between abnormal leukocyte counts and mortality may reflect infection, systemic inflammation, or other manifestations of advanced liver disease.

Elevated serum creatinine was another factor significantly associated with mortality. Renal dysfunction is a frequent complication of decompensated cirrhosis, including hepatorenal syndrome. Musunuri et al. and Jun et al. reported that serum creatinine ≥ 2.0 mg/dL is strongly associated with short-term mortality in cirrhotic patients, supporting our findings [4,5]. Renal dysfunction reflects systemic circulatory impairment and is a key component of prognostic scoring systems such as MELD, which are widely used to predict mortality in cirrhotic patients [11].

Elevated total bilirubin was also significantly associated with mortality. Bilirubin reflects hepatic excretory function, and its elevation indicates advanced hepatocellular dysfunction. This finding is consistent with previous studies demonstrating that hyperbilirubinemia is strongly associated with disease severity and mortality risk, and it is incorporated into prognostic scoring systems such as MELD [11].

High INR was strongly correlated with mortality in this study. INR reflects hepatic synthetic function for coagulation, and elevated values indicate advanced hepatocellular dysfunction. Studies by Duah et al. from Ghana and Ameena et al. from India also reported INR ≥ 1.6 as a factor associated with in-hospital mortality in cirrhotic patients [7,12].

Hyponatremia emerged as another important factor. Low serum sodium reflects circulatory and hemodynamic disturbances associated with portal hypertension and neurohumoral activation. Previous studies have shown that each 1 mmol/L reduction in serum sodium increases mortality risk by up to 5% [13,14]. Hyponatremia is also incorporated into modified prognostic models such as MELD-Na, highlighting its importance in predicting mortality in patients with advanced liver disease [15].

In addition to individual laboratory parameters, several prognostic scoring systems, such as Child-Pugh, MELD/MELD-Na, and ALBI scores, have been developed to support mortality assessment in cirrhotic patients. These scoring systems integrate multiple clinical and biochemical variables and have been shown to predict mortality more accurately than single parameters alone [15]. Conversely, age, sex, comorbidities, and upper gastrointestinal bleeding at admission were not significantly associated with mortality.

This study provides real-world data from a tertiary referral hospital in Indonesia and specifically reflects outcomes among hospitalized patients with decompensated liver cirrhosis. Several clinically relevant and routinely available clinical and laboratory parameters were evaluated, which may assist clinicians in identifying patients at higher risk of adverse in-hospital outcomes, particularly in resource-limited settings.

This study was intended as an exploratory analysis of clinical and laboratory factors associated with in-hospital mortality among hospitalized patients with decompensated liver cirrhosis. Although several variables showed significant associations with mortality, multivariate analysis was not performed; therefore, independent predictors and causal relationships could not be established. The findings should therefore be interpreted as associative rather than predictive. A further limitation of this study is that the standardized criteria required to diagnose acute-on-chronic liver failure (ACLF) were not consistently available in the retrospective dataset. Consequently, patients with ACLF could not be reliably identified or analyzed separately.

This study has several limitations. First, the retrospective design may introduce bias due to incomplete medical records. Second, the exclusion of patients with incomplete data may have resulted in selection bias, as these patients might have had less severe disease and better outcomes, potentially leading to an overestimation of mortality rates. Third, this study was conducted in a single center, which may limit the generalizability of the findings. Future studies using multivariate models are recommended to identify independent predictors and validate these findings.

## Conclusions

This study demonstrated that in-hospital mortality among patients with decompensated liver cirrhosis at a tertiary referral hospital in Indonesia remains high. Hepatic encephalopathy, leukocyte abnormalities, hypoalbuminemia, elevated bilirubin, elevated creatinine, increased INR, hyponatremia, and higher prognostic scores were significantly associated with mortality. Early recognition of these factors may help clinicians identify patients at higher risk of adverse in-hospital outcomes.
